# Cutaneous Manifestations and Neurological Diseases

**DOI:** 10.7759/cureus.47024

**Published:** 2023-10-14

**Authors:** Arpita Lahoti, Adarshlata Singh, Yuganshu T Bisen, Amey M Bakshi

**Affiliations:** 1 Department of Dermatology, Venereology and Leprosy, Jawaharlal Nehru Medical College, Datta Meghe Institute of Higher Education and Research, Wardha, IND

**Keywords:** skin, neurology, cutaneous, genodermatoses, neurocutaneous

## Abstract

Our skin and nervous system are tightly connected. Numerous dermatomes on our skin provide sensory information to the brain. Because skin changes can occasionally be the first sign of a neurological problem, understanding skin alterations is crucial as it can indicate early about the underlying condition, which can affect the prognosis of the disease. In these cases, the dermatologists' and neurologists' skills are complementary to each other. In this article, we have categorized diseases with neuro-cutaneous manifestations under different headings, such as infections, metabolic diseases, connective tissue disorders, genodermatoses, nutritional deficiency, and the diagnostic criteria of some commonly encountered diseases. Through tabulation, it has been observed that this categorization can serve as a useful reference for managing day-to-day patients who are either diagnosed with the diseases mentioned above or suspected to have the conditions.

## Introduction and background

The central nervous system and skin are intimately linked. There are many diseases which involve the nervous system and the skin simultaneously. In these cases, the dermatologists' and neurologists' skills are complementary to each other. The neural crest gives rise to melanocytes, an essential skin component, and has similar embryonic origins to the nerve tissues found in the brain and spinal cord. Cutaneous dermatomes receive sensory input from C fibers, alpha fibers, beta fibers, and delta fibers, which end in the sensory apparatus of the skin. The eccrine sweat gland, hypodermic vascular tone, and piloerection are all under the control of sympathetic and parasympathetic nerves. Numerous central and peripheral nervous disorders have cutaneous symptoms of neurology and neuroendocrinology that are clinically relevant and may be physiologically significant [1-4].

## Review

Methodology

**Figure 1:**
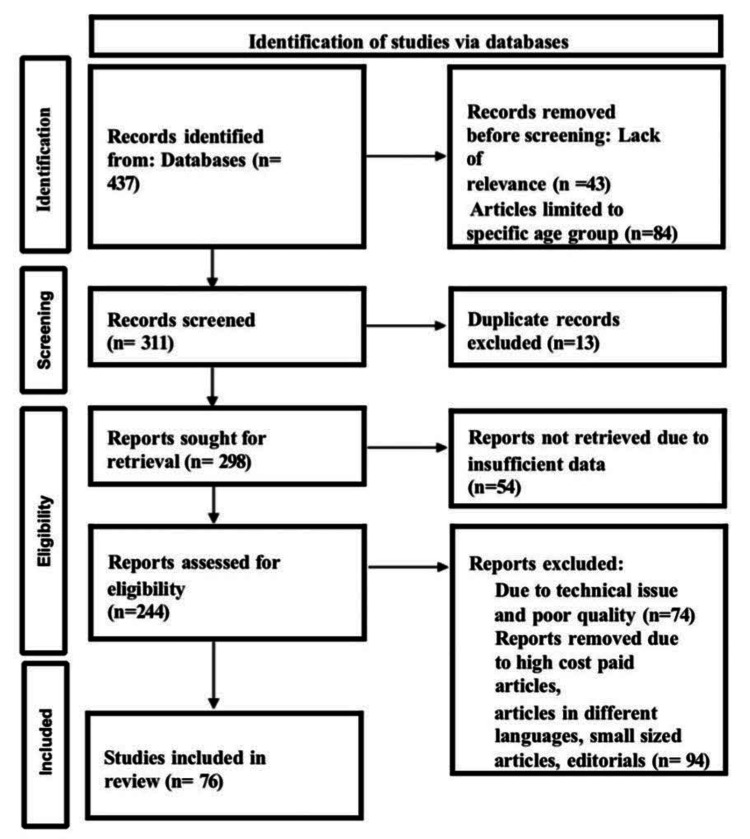
PRISMA model for search strategy. PRISMA: Preferred Reporting Items for Systematic Reviews and Meta-Analyses.

The following keywords were used in Embase, Scopus, Cochrane, Google Scholar, and advanced PubMed searches: cutaneous, genodermatosis, neurocutaneous, and neurological disorders. The search yielded 437 articles, of which 76 research publications were selected for research. The methodology of the Preferred Reporting Items for Systemic Reviews and Meta-Analyses (PRISMA) method is shown in Figure 1. Genodermatoses, such as neurofibromatosis, tuberous sclerosis, and ataxia-telangiectasia, may present cutaneous manifestations followed by systemic manifestations. This necessitates that neurologists, physicians, and dermatologists be well-versed in these conditions. Upon reviewing the literature, we attempted to categorize a few common conditions into groups: infections, metabolic diseases, connective tissue disorders, genodermatoses, and nutritional deficiencies. These categorizations are as follows: a) Infections, cutaneous and neurological manifestations, b) Metabolic diseases, cutaneous and neurological manifestations, c) Connective tissue disorders, cutaneous and neurological manifestations, d) Genodermatoses, Café au lait cutaneous lesions and neurological manifestations, e) Nutritional deficiencies, cutaneous and neurological manifestations.

As a dermatologist, the disorders encountered daily in the OPD are listed below. These can be diagnosed through their clinical features and additional diagnostic methods, as outlined below.

Herpes Zoster

Clinical signs of herpes zoster include burning pain, distinct morphology, and typical distribution [5-6]. Various tests for the varicella-zoster virus are utilized, including detecting IgM antibodies specific to varicella-zoster in the blood and observing multinucleated giant cells on the Tzanck smear of vesicular fluid. Additional methods involve polymerase chain reaction testing of vesicular fluid and direct fluorescent antibody testing [7-11].

Leptospirosis

Skin manifestations in leptospirosis largely result from vasculitis, where the inflammation of small blood vessels precipitates skin hemorrhages and petechiae. Diagnosis might involve a variety of tests; the microscopic agglutination test is one primary method. Further, comprehensive blood work may encompass renal and liver function tests, a complete blood count, and coagulation studies. Cerebrospinal fluid can be analyzed, typically obtained via a lumbar puncture, especially when aseptic meningitis is suspected in the immunological phase. Additionally, due to the potential involvement of numerous organ systems, chest X-rays might also be requisitioned to provide a thorough assessment of the patient's condition [12-20].

Syphilis

Dark-field microscopy and serological tests are utilized for diagnosing syphilis, with the latter being bifurcated into treponemal and non-treponemal types. The non-treponemal assays, such as the rapid plasma reagin test (RPR) and venereal disease research laboratory test (VDRL), are screening tests that look for antibodies to cardiolipin in blood but lack specificity for syphilis. A treponemal test that identifies particular serum antibodies to Treponema pallidum must be used to confirm positive non-treponemal testing. Notable examples of these tests include the fluorescent treponemal antibody absorption assay (FTA-ABS) and *Treponema pallidum* particle agglutination assay (TP-PA) [21-27].

Tuberculosis

The Mantoux test, involving skin testing with PPD, is a customary diagnostic approach for tuberculosis. In all cases when a screening test is positive, a chest x-ray is advised to either rule out or confirm the existence of active illness. Additional diagnostic and confirmatory tests, such as Ziehl-Neelsen Acid Fast Staining and culture, are also pivotal. The advent of Nuclear Amplification and Gene-Based Tests, which employ Deoxyribonucleic acid-based molecular techniques to detect the bacteria or its particles, marks the latest advancements in tuberculosis testing. Notable among these are the GeneXpert and tests for Drug-resistant Mycobacterium tuberculosis. The newest molecular-based procedures are quicker and allow for precise, quick diagnosis. According to histopathology, the granuloma is recognized as the histological hallmark of tuberculosis [27-35].

Primary Amyloidosis

Clinical suspicion, familial history, and tissue biopsy are foundational in establishing a diagnosis of primary amyloidosis [36-41].

Diabetes Mellitus

The American Diabetes Association (ADA) establishes that a diabetes diagnosis can be confirmed through any of the following criteria: an HbA1c (glycated hemoglobin) level of 6.5% or higher; a fasting plasma glucose level of 126 milligrams per deciliter (mg/dL) or 7.0 millimoles per liter (mmol/L) or greater, ensuring no caloric intake for at least eight hours; a 75-gram oral glucose tolerance test revealing a two-hour plasma glucose level of 200 mg/dL (11.1 mmol/L) or higher. Additionally, a patient exhibiting symptoms of hyperglycemia (such as excessive thirst, frequent urination, insatiable hunger, and weight loss) or undergoing a hyperglycemic crisis, with a random plasma glucose measurement of 200 mg/dL (11.1 mmol/L) or above, can be diagnosed with diabetes [42].

Arsenic Poisoning

A history of acute gastroenteritis followed by hypotension, as well as a risk of occupational or environmental hazards, should make practitioners more cautious. Signs of persistent exposure may include hyperpigmentation, peripheral neuropathy, or recurrent gastroenteritis. A higher-than-normal arsenic level in a 24-hour urine collection is the best sign of arsenic exposure. Urine testing on the spot is potential in emergencies. Arsenic exposure is considered to have occurred at a concentration of 50 micrograms/liter or more. In cases of unknown chronic exposure, hair and nail clippings should be collected for laboratory investigation [43-47].

Systemic Lupus Erythematosus

Manifestations of systemic lupus erythematosus are due to immune complex deposition and subsequent inflammation. Diagnosis and classification of SLE typically require meeting at least four out of the eleven criteria stipulated by the 1997 American College of Rheumatology. It is identified based on a combination of signs and symptoms, indicators, and relevant laboratory tests. Additionally, histology and imaging are important. Key indicators can include malar rash, Raynaud's phenomenon, photosensitivity, oral/nasal ulcers, discoid rash, alopecia, non-erosive arthritis in two or more peripheral joints, and serositis (pleura or pericardium inflammation). Additionally, renal disease (evidenced by daily proteinuria exceeding 500 milligrams or cellular casts), hematologic abnormalities (such as a platelet count under 100,000/cubic millimeter in the absence of platelet-lowering drugs, or a white blood cell count under 4,000/cubic millimeter on two or more occasions), seizures, and psychosis without alternative explanation can also be indicative. Immunologic conditions might involve the presence of antiphospholipid antibodies, determined by either an abnormal serum level of IgM or IgG anticardiolipin antibodies, a positive test result for lupus anticoagulant, anti-deoxyribonucleic acid antibody, or anti-Smith antibody, or a false-positive syphilis test using the Venereal Disease Research Laboratory test or rapid plasma reagin. Moreover, antinuclear antibody positivity must be established in the absence of medications known to induce drug-induced lupus [48-54].

Rheumatoid Arthritis

The 2010 diagnostic criteria for rheumatoid arthritis, outlined by the American College of Rheumatology and the European League Against Rheumatism (ACR/EULAR), encompass four distinct domains. The size of the affected joints: one to three small joints equal to two points (metacarpophalangeal joints, second to fifth metatarsophalangeal joints, proximal and thumb interphalangeal joints, and wrists), four to ten small joints equal to three points (ankles, knee, hip, shoulder, elbow), more than 10 joints, plus at least one minor joint, equals five points. Serological testing for anti-citrullinated peptide/protein antibodies or rheumatoid factor is assigned two points for a low positive and three points for a high positive. Raised erythrocyte sedimentation rate (ESR) or C-reactive protein (CRP) equals one point. Lastly, a minimum of six weeks of symptoms equals one point. The patient is diagnosed as having rheumatoid arthritis if their total score is equal to or greater than six [54-65].

Neurofibromatosis 1

The abnormal growth of neural crest-derived cells is thought to be the cause behind these manifestations of neurofibromatosis 1. To diagnose according to the accepted clinical National Institutes of Health (NIH) criteria, at least two or more of the following conditions must be met: six or more café-au-lait macules, ranging from five to fifteen millimeters in maximum diameter; at least one plexiform neurofibroma or two or more neurofibromas of any type; freckling in the inguinal or axillary regions; optical gliomas; a minimum of two Lisch nodules; bony lesions, such as pseudoarthrosis or sphenoid dysplasia; and a first-degree relative with the condition [66]. The following is a list of the Revised Diagnostic Criteria issued in 2021. The following should be present in a patient without any family history in two or more cases: six or more café au lait macules measuring over 5 mm before puberty and over 15 mm after; freckling in the inguinal or axillary regions; one plexiform neurofibroma or two or more neurofibromas of any type; an optic nerve pathway tumor; two or more iris choroidal anomalies; a distinct osseous condition, like pseudoarthrosis of a long bone, tibial bowing, or sphenoid dysplasia; or a hazardous Neurofibromatosis 1 variant. For a child with a supportive family history, only one or more of the conditions in section A need to be present [67-71].

Pellagra

To validate the findings, laboratory testing, including measurements of tryptophan, nicotinamide adenine dinucleotide phosphate, and niacin levels, should be carried out. The diagnosis of pellagra is based on the patient's therapeutic response to niacin treatment and the characteristic symptoms [72].

Vitamin B12

A complete blood count, along with a peripheral smear, serum B12, and serum folate levels, serve as indicators for vitamin B12. Once a B12 deficiency is confirmed, its cause must be identified and addressed. In cases of B12 insufficiency, serum methylmalonic acid and serum homocysteine should be elevated. Moreover, these laboratory findings can assist in differentiating between a folate deficiency and a B12 deficiency. In the context of folate insufficiency, homocysteine levels are elevated, while methylmalonic acid levels remain normal [73-76]. All cutaneous and neurological manifestations are summarized in Table 1.

**Table 1:** Summarizing all the cutaneous and neurological findings due to infections, metabolic diseases, connective tissue disorders, genodermatoses, and nutritional deficiencies.

S no.	Author	Year	Study design	Disease	Basic lesion	Cutaneous manifestation	Neurological manifestation	
1	Hurko O and Provost TT [2]	1999	Original research	Brill-Zinser epidemic typhus, Herpes varicella zoster, Coccidiomycosis, Chagas disease ,Haemophilus influenza, Infantile multisystem inflammatory disease, Leptospirosis, listeria, Lyme borreliosis, Meningococcaemia, Familial histiocytic Rocky mountain spotted fever, Syphilis, Tuberculosis, Varicella-zoster (chickenpox), Yersinia pestis (bubonic plague), Amyloidosis (primary), Arsenic poisoning, Chediak-Higashi, Diabetes mellitus, Dysautonomia, familial, Fabry Disease, Flynn-Aird Syndrome, Linear sebaceous nevi of Jadassohn, POEMs syndrome, Adult Refsum’s disease, Thallium intoxication, Xeroderma pigmentosum, Systemic lupus erythematosus, Sarcoidosis, Sjögren’s syndrome, Neurofibromatosis type 1, Ataxia telangiectasia variant VI, Russell-Silver dwarfism, Pellagra, Vitamin B12 deficiency	Macule, Vesicles and bulla, Nodules and sinus, Target lesions or erythema multiforme, Nodular, Maculo papular rash, Maculopapular rash, Macule (petechiae), papule, Macule and papule, Ulcer, scales, plaque and warty appearance and EM, Macule (purpura) and bullous lesions, Macular and scaly lesions, Generalised Hypo -pigmentation, Paulo-nodular and ulcerative lesions, Cutaneous Hypo- Hyper pigmentation, Purplish macular lesions, Ectodermal characteristics, Cutaneous atrophy and ulcerations Plaque, Macular Hyperpigmentation, Scaly Plaque, Atrophy, teleangiectasia, Erythematous Macular lesions, nodules, Papule, maculopapular & Subcutaneous nodular, Erythematous macular lesions, Hyperpigmented macules/patches.	Macular rash, oral lesions with vesicles, Erythema nodosum, draining sinus, subcutaneous cellulitis, Romana’s sign, lacrimal gland inflammation, erythema multiforme, Typically indurated area found on the arm, face, and upper chest, neck, Evanescent rash, inflammation of uvea, Scleral conjunctival injection, rash having macules and papules on trunk and jaundice, Animal specialist with tender red papules of hands, Generalised erythematous papules, petechiae in infants, Target lesion, Small, uneven, smudged petechiae that are typically found on the extremities and trunk; at first glance, they may resemble a viral exanthem, Pinkish macules on the wrists, ankles, and forearms on the fourth day of fever indicate the beginning of the usually developed rash. After six to eighteen hours, the palms soles are involved, then centrally after one-three days deep red macules, and finally non-blanching petechiae after two-four days follow, Primary: chancre; secondary; non-itchy rash with macules and papules (acral), patchy alopecia, erythema multiforme, condyloma lata hyperpigmentation after healing, mucous patches., and lesions on the palms and soles, Cutaneous tuberculosis is rare; post-primary lupus vulgaris, scrofuloderma, erythema nodosum and multiforme, warty tuberculosis verrucosa cutis from re-infection, Oral lesions with vesicles, Erythema multiforme (target lesions), bubos followed by ecchymoses petechiae, Bullae on the skin or oral mucosa, papules, occasionally alopecia, and skin folds or flat surfaces covered in purpura, Mees lines, linear nail hyperpigmentation, dry scaly desquamation, etc, Partial albinism, silvery blond hair, Necrobiosis lipoidica diabeticorum, poorly healing ulcer, Blotching, abnormal sweating, hypohidrosis, Angiokeratomas mainly distributed over trunk region, skin atrophy, alopecia, dental caries hyperkeratosis, chronic ulceration, sebaceous hyperplasia and epithelial nevi, Cutaneous thickening, sclerodermoid changes, Hypertrichosis, angiomas, Terry’s nail, hyperhidrosis and Raynaud’s phenomenon, Ichthyosis, Early-onset basal and squamous cell, malignant melanoma skin cancers, sensitivity to light, atrophy, angioma, telangiectasia, actinic keratosis, and keratoacanthoma, Photosensitivity, malar rash, telangiectasia, discoid lupus, patchy alopecia, mucosal ulcers, angioneurotic oedema, Raynaud’s phenomenon, subcutaneous nodules, palpable purpura, gangrene, erythema multiforme (rare), Hypohidrosis, cicatricial alopecia; acute: erythema nodosum, vesicles, maculopapular rash; chronic lupus pernio, plaques, scars, keloids Purpura, Raynaud’s phenomenon, xerostomia, candidiasis, dental caries ,keratoconjunctivitis sicca, café au lait macules, café au lait spots, Erythematous photosensitive rash, erythema, vesicles, glossitis, malar and supraorbital hyperpigmentation, rhagades, Black nail pigmentation (nail bed and matrix), oral aphthae.	Meningo-encephalitis, Meningitis; maybe from para meningeal focus in case of osteomyelitis of vertebrae, Encephalitis, Acute purulent meningitis, Papilledema, aseptic meningitis, optic, atrophy, mental disability, Meningitis with subacute onset, Meningitis with subacute onset, Early aseptic meningitis, polyneuropathy, delayed demyelinating disease, Fulminant meningitis, Vasculitic meningoencephalitis, deafness, choreoathetosis, hemiplegia, Aseptic meningitis is seen in secondary phase; late meningovascular syphilis; tabes dorsalis, CNS tuberculomas, Pott’s disease, Chronic meningitis, ataxia and meningitis, All three types— bubonic-septicaemic, bubonic, pneumonic can be complicated by meningitis, peripheral neuropathy, Chronic meningitis with cranial neuropathies, distal neuropathy and proximal myopathy; leukopathy, hypothalamic involvement and hyperprolactinemia [2] Aseptic meningitis, dorsal ganglionopathy with sensory ataxia, dural sinus thrombosis , peripheral neuropathy	
2	Hanna GR [3]	1918	Original article	Herpes varicella zoster	Vesicles and bulla	No cutaneous manifestations	cerebellar ataxia, meningitis	
3	Yeung C and Baranchuk A [4]	2018	Review	Lymes disease	Macular and papular	acrodermatitis chronica atrophicans, Erythema migrans	Headache, facial palsy, Inflammation of brain and spinal cord, Nerve pain	
4	Paparone P and Paparone PW [5]	2018	Systematic review	babesiosis	Macular and papular	acrodermatitis chronica atrophicans, Erythema migrans	Headache, facial palsy, Inflammation of brain and spinal cord, Nerve pain	
5	Trayes KP et al. [6]	2018	Review Article	Annular Lesions	Macular and papular	acrodermatitis chronica atrophicans, Erythema migrans	Headache, facial palsy, Inflammation of brain and spinal cord, Nerve pain	
6	Maurer MS et al. [17]	2017	Review Article	Cardiac Amyloidosis	Macule (purpura) and bullous lesion	No cutaneous manifestations	peripheral neuropathy, may present as Alzheimer's disease	
7	Pinney JH et al. [18]	2013	Research Article	Senile systemic amyloidosis	Macule (purpura) and bullous lesion	No cutaneous manifestations	peripheral neuropathy, may present as Alzheimer's disease	
8	Abernathy CO and Ohanian EV [19]	1992	Review Article	Arsenic posioning	Macular and scaly lesions	No cutaneous manifestations	Acute: headache, delirium, encephalopathy, and seizures. Subacute: Reversible sensorimotor polyneuropathy	
9	Beckman KJ et al. [20]	1991	Case Report	Arsenic posioning	Macular and scaly lesions	No cutaneous manifestations	Acute: headache, delirium, encephalopathy, and seizures. Subacute: Reversible sensorimotor polyneuropathy	
10	Bolliger CT et al. [21]	1992	Case Report	Arsenic posioning	Macular and scaly lesions	No cutaneous manifestations	Acute: headache, delirium, encephalopathy, and seizures. Subacute: Reversible sensorimotor polyneuropathy	
11	Chhuttani PN et al. [22]	1967	Research Article	Arsenic posioning	Macular and scaly lesions	No cutaneous manifestations	Acute: headache, delirium, encephalopathy, and seizures. Subacute: Reversible sensorimotor polyneuropathy	
12	Unger RH and Orci L [23]	2010	Perspective	Diabetes mellitus	Paulo-nodular and ulcerative lesions	No cutaneous manifestations	peripheral neuropathy	
13	Walker HK et al. [24]	1976	Review Article	Dysautonomia, familial	Cutaneous Hypo- Hyper - pigmentation	No cutaneous manifestations	peripheral neuropathy	
14	Mutoh T et al. [25]	1988	Case Report	Fabry Disease	Purplish macular lesions	No cutaneous manifestations	Small fibre neuropathy, autonomic neuropathy	
15	NIH [26]	2023	Genetic and Rare Disease Information Centre	Flynn-Aird Syndrome	Ectodermal characteristics Cutaneous atrophy and ulcerations	No cutaneous manifestations	Dementia, epilepsy, ataxia and peripheral neuropathy	
16	Pauline L et al. [27]	2014	Case Report	Linear sebaceous nevi of Jadassohn	Plaque	No cutaneous manifestations	Mental retardation, CNS abnormalities	
17	Sarwar M and Schafer ME [28]	1988	Case Report	Linear sebaceous nevi of Jadassohn	Plaque	No cutaneous manifestations	Mental retardation, CNS abnormalities	
18	Brown R and Ginsberg L [29]	2019	Clinical Update	POEMs syndrome	Macular Hyperpigmentation	No cutaneous manifestations	Polyneuropathy	
19	Koike H et al. [30]	2008	Research Paper	POEMs syndrome	Macular Hyperpigmentation	No cutaneous manifestations	Polyneuropathy	
20	Liu M et al. [31]	2015	Research Paper	POEMs syndrome	Macular Hyperpigmentation	No cutaneous manifestations	Demyelination and axonal degeneration	
21	Bamiou DE et al. [32]	2003	Case Report	Adult Refsum’s disease	Scaly Plaque	No cutaneous manifestations	Demyelination and axonal degeneration	
22	Vandana VP et al. [33]	2015	Case Report	Adult Refsum’s disease	Scaly Plaque	No cutaneous manifestations	asymmetric polyneuropathy	
23	Harari D et al. [34]	1991	Research Article	Adult Refsum’s disease	Scaly Plaque	No cutaneous manifestations	asymmetric polyneuropathy	
24	Osorio-Rico L et al. [35]	2017	Neurotoxicity of Metals pp 345-353	Thallium intoxication	Cutaneous Atrophy	Alopecia, Mee’s lines	peripheral neuropathy, cranial nerve palsies, ataxia	
25	Sojáková M et al. [36]	2015	Case Report	Thallium intoxication	Cutaneous Atrophy	Alopecia, Mee’s lines	peripheral neuropathy, cranial nerve palsies, ataxia	
26	Xiao T et al. [37]	2012	Review Article	Thallium intoxication	Cutaneous Atrophy	Alopecia, Mee’s lines	peripheral neuropathy, cranial nerve palsies, ataxia	
27	Neill CA and Dingwall MM [38]	1950	Review of Cases	Xeroderma pigmentosum	Atrophy, teleangiectasia	No cutaneous manifestations	Neurodevelopment deficiency or sensorineural deafness., microcephaly, ataxia	
28	Schmickel RD et al. [39]	1977	Case Report	Xeroderma pigmentosum	Atrophy, teleangiectasia	No cutaneous manifestations	Neurodevelopment deficiency or sensorineural deafness., microcephaly, ataxia	
29	Ruacho G et al. [45]	2022	Original Research	Systemic lupus erythematosus	Erythematous Macular lesions, nodules	No cutaneous manifestations	Headache, Seizures, psychosis, aseptic meningitis, demyelinating syndrome, cranial and peripheral neuropathies, mononeuritis multiplex, Myasthenia gravis	
30	Casey AT et al. [46]	1997	Journal of Neurosurgery Volume 87: Issue 6 Page Range: 856-862	Rheumatoid arthritis	Neurocutaneous disorders in Connective tissue diseases	subcutaneous nodules at sites of trauma: extensor surfaces of forearms, ears, and posterior scalp	high cervical myelopathy, atlantoaxial instability	
31	Said G [47]	1997	Neurologic Clinics: Volume 15,Issue 4	Rheumatoid arthritis	Neurocutaneous disorders in Connective tissue diseases	No cutaneous manifestations	mononeuritis multiplex	
32	Upadhyaya M et al. [50]	2007	Article	Neurofibromatosis type 1	macules	No cutaneous manifestations	optic pathway glioma, plexiform neurofibroma, Astrocytomas, ependymomas and medulloblastomas, megalencephaly, scoliosis	
33	Pollack IF and Mulvihill JJ [51]	1997	Brain Pathology: Volume 7, Issue 2	Neurofibromatosis type 2	macules	cafe au lait spots, dermal neurofibromas	No neurological manifestations	
34	Gutmann DH et al. [52]	1997	Review Article	Neurofibromatosis type 2	macules	No cutaneous manifestations	bilateral vestibular schwannomas, meningioma, glioma, schwannoma, and juvenile posterior subcapsular lenticular opacity/juvenile cortical cataract	
35	Webb DW et al. [53]	1996	Review Article	Tuberous sclerosis	macules	adenoma sebaceum, periungual angiokeratomas, Shagreen patches, hypopigmented ash leaf spots	No neurological manifestations	
36	Shepherd CW et al. [54]	1991	comparative study	Tuberous sclerosis	macules	No cutaneous manifestations	severe mental retardation and infantile spasms, ganglioneuromas	
37	Weemaes CM et al. [55]	1981	Acta Paediatrica/Volume 70, Issue 4/p. 557-564	Ataxia telangiectasia variant VI	macules	No cutaneous manifestations	microcephaly with (usually) normal intelligence, immunodeficiency	
38	Smith DW [56]	1976	Article	Bloom’s syndrome	macules	Facial telangiectasias	No neurological manifestations	
39	Hagerman DA, Williams GP [57]	1993	Images in Clinical Medical	Bloom’s syndrome	macules	No cutaneous manifestations	dolichocephaly and light sensitivity, mild learning disability	
40	Tisherman SE et al. [58]	1962	Article	Familial Pheochromocytoma	macules	cafe au lait spots as well as hypopigmented patches	hemangioblastomas of the cerebellum and spinal cord, retinal angiomas	
41	Tisherman SE et al. [59]	1993	Case Report	Von Hippel-Lindau syndrome	macules	cafe au lait spots as well as hypopigmented patches	hemangioblastomas of the cerebellum and spinal cord, retinal angiomas	
42	Hamilton SR et al. [60]	1995	Original Article	Turcot’s syndrome	macules	café au lait spots	No neurological manifestations	
43	Hamosh A et al. [61]	2000	Review Article	Turcot’s syndrome, McCune Albright polyostotic fibrous dysplasia, Russell-Silver dwarfism	macules	café au lait spots	brain tumours, usually medulloblastomas, colon cancer associated with polyposis, thyroid carcinoma, and bone cysts. brainstem compression and syringomyelia resulting from severe basilar invagination, Microcephaly but usually normal intelligence	
44	Endo M et al. [62]	1991	Article	McCune Albright polyostotic fibrous dysplasia	macules	Very large, unilateral and segmental café au lait spots	No neurological manifestations	
45	Hurst JA et al. [63]	2005	Review Article	McCune Albright polyostotic fibrous dysplasia	macules	No cutaneous manifestations	brainstem compression and syringomyelia resulting from severe basilar invagination	
46	Westerhof W et al. [64]	1978	Case Report	Westerhof syndrome	Hypo-hyper pigmented macules/patches	congenital hypopigmented and hyperpigmented patches, café au lait spots[	mental retardation	
47	Patton MA [65]	1988	Original Article	Russell-Silver dwarfism	Hypo-hyper pigmented macules/patches	No cutaneous manifestations	Microcephaly but usually normal intelligence	
48	Suter PM and Russell RM [68]	2018	Harrison's Principles of Internal Medicine, 20e: Chapter 326	Pellagra	Macules	Casal’s necklace-erythematous scaly. well defined plaques over sun exposed sites,stomatitis, glossitis	Pellagrous encephalopathy – apathy, memory loss, disorientation, Depression or delirium	
49	MacDonald A and Forsyth A [69]	2005	Review Article	Pellagra	Macules	Casal’s necklace-erythematous scaly. well defined plaques over sun exposed sites,stomatitis, glossitis.	Pellagrous encephalopathy – apathy, memory loss, disorientation, Depression or delirium	
50	Oldham MA and Ivkovic A [70]	2012	Case Study	Pellagra	Macules	Casal’s necklace-erythematous scaly. well defined plaques over sun exposed sites,stomatitis, glossitis.	Pellagrous encephalopathy – apathy, memory loss, disorientation, Depression or delirium	
51	Pipili C et al. [71]	2008	Case Report	Pellagra	Macules	Casal’s necklace-erythematous scaly. well defined plaques over sun exposed sites,stomatitis, glossitis.	Pellagrous encephalopathy – apathy, memory loss, disorientation, Depression or delirium	
52	Oo TH and Rojas-Hernandez CM [72]	2017	Review Article	Vitamin B 12 deficiency	Macules	No cutaneous manifestations	Neurologic deficit-neuropathy, ataxia, subacute combined degeneration of the spinal cord -peripheral neuropathy and dementia	
53	Cavalcoli F et al. [73]	2017	Review Article	Vitamin B 12 deficiency	Macules	No cutaneous manifestations	Neurologic deficit-neuropathy, ataxia, subacute combined degeneration of the spinal cord -peripheral neuropathy and dementia	

## Conclusions

The article explores the intricate relationship between the skin and the nervous system. It underscores the significance of recognizing dermatological changes as potential indicators of underlying neurological conditions. This understanding is paramount for early diagnosis and prognosis, bridging the expertise of dermatologists and neurologists. The review methodically categorizes diseases with neuro-cutaneous manifestations, encompassing infections, metabolic disorders, connective tissue abnormalities, genodermatoses, and nutritional deficiencies. This categorization provides a valuable reference tool for healthcare professionals dealing with patients exhibiting symptoms in these domains. However, it is worth noting that while this article provides a solid foundation for understanding neuro-cutaneous diseases, further research and clinical studies are needed to expand our knowledge in this field. More detailed insights into the mechanisms underlying these conditions, as well as advancements in diagnostic tools and treatment options, would enhance the depth of this article. Furthermore, the article delves into the diagnostic criteria for several commonly encountered diseases. It elucidates the diagnostic processes for conditions such as herpes zoster, leptospirosis, syphilis, tuberculosis, primary amyloidosis, diabetes mellitus, arsenic poisoning, systemic lupus erythematosus, rheumatoid arthritis, neurofibromatosis 1, pellagra, and vitamin B12 deficiency. While these diagnostic criteria serve as valuable guidelines, ongoing research may refine and expand upon these parameters, leading to even more accurate and efficient diagnoses in the future. In essence, this article serves as a strong foundation for understanding the intersection of dermatology and neurology. However, it also highlights the need for continuous research and clinical advancements to improve our knowledge and ultimately enhance patient care in this critical medical field.
